# Genome editing around the globe: An update on policies and perceptions

**DOI:** 10.1093/plphys/kiac359

**Published:** 2022-08-17

**Authors:** Thorben Sprink, Ralf Wilhelm, Frank Hartung

**Affiliations:** Julius Kuehn Institute (JKI) – Federal Research Centre for Cultivated Plants, Institute for Biosafety in Plant Biotechnology, Quedlinburg, Saxony-Anhalt, Germany; Julius Kuehn Institute (JKI) – Federal Research Centre for Cultivated Plants, Institute for Biosafety in Plant Biotechnology, Quedlinburg, Saxony-Anhalt, Germany; Julius Kuehn Institute (JKI) – Federal Research Centre for Cultivated Plants, Institute for Biosafety in Plant Biotechnology, Quedlinburg, Saxony-Anhalt, Germany

## Abstract

A decade ago, the CRISPR/Cas system has been adapted for genome editing. Since then, hundreds of organisms have been altered using genome editing and discussions were raised on the regulatory status of genome edited organisms esp. crops. To date, many countries have made decisions on the regulatory status of products of genome editing, by exempting some kinds of edits from the classical GMO regulation. However, the guidance differs between countries even in the same region. Several countries are still debating the issue or are in the progress of updating guidance and regulatory systems to cover products of genome editing. The current global situation of different regulatory systems is putting a harmonized framework on genome-edited crops in the far future. In this update, we summarize the current developments in the field of regulation concerning edited crops and present a short insight into perception of genome editing in the society.

## Teaser

With this updated review, we give an update on the current regulatory and political developments of genome editing and its products around the globe.

**ADVANCES**

- Recent developments and updates in the regulatory regimes for new breeding technologies and related products have been published. Here, we present a short and easy-to-read update on the recent developments in the legislation in England, Canada, Nigeria, Kenya, South Africa, Japan, India, and China.
- We highlight the recent developments in discussions concerning NBTs in countries that have not released any formal legislation yet, e.g. Europe, Switzerland, South Korea, Indonesia, The Philippines, Vietnam, Taiwan, and Hungary.
- Looking at social perception of GE, we present a summary of late consumer and stakeholder surveys from Scandinavia and England that, in contrast to elder surveys, show a more distinguished attitude of the participants regarding food and feed produced by GE.

Before Mendel’s laws shed light on genetic processes plant breeding was unsystematically choosing and selecting plants for propagation that showed (a combination of) preferential traits. Outcomes were highly variable and improvements took considerable time. The transfer of a trait of interest from a preselected donor line through systematic crossing to a target variety is, nevertheless, challenged by mingling the whole genomes of both parents, and the accessible variation of traits is limited to crossable relatives. With the use of radiation or chemicals for mutation-induction, variation in the genome could be actively created. However, the changes occur randomly and thus are still undirected and multiple. Even with the possibility to confer genes from a related or unrelated organism into the genome by genetic engineering, the changes are still undirected but unwanted additional changes could be limited. In the last decade, things changed, with the development of new breeding techniques (NBTs) such as genome editing (GEd) as a tool for plant breeding. Precise and site-directed changes in the genome have become possible in a rather easy way in many plant species (Modrzejewski et al., 2019). GEd has made breeding more predictable and paved the way for a timelier adaption of crop plants to a changing environment and new challenges.

GEd uses either variants of side-directed nucleases (SDNs) or oligo-nucleotide-directed mutagenesis or a combination of both. Application of GEd techniques can result in changes in the genome that are comparable to those achieved with conventional breeding or already established mutation techniques but with a reduced number of side effects such as unwanted additional mutations (Off-Targets; Modrzejewski et al., 2020). However, GEd can also introduce allochthonous sequences into the genome just as classical transgenesis or autochthonous sequences as in cisgenesis at a predefined site in a genome. Currently, four SDN systems have been adapted to be used as genome editors in plants: (1) Zinc-finger nucleases and (2) Meganucleases, which emerged in the last years of the last century, but have only a limited relevance for the field of GEd in plants (Modrzejewski et al., 2019); (3) TALE-nucleases, which enabled a single nucleotide accuracy (Becker and Boch, 2021) and paved the way for a broader use of GEd techniques in plant science as well as in breeding, and (4) the most recent branch of GEd techniques based on CRISPR-systems and derivatives. The CRISPR systems employ mechanisms adapted from bacteria and archaea, which use these for a kind of adaptive defense system against invading nucleic acids (DNA and RNA). Since their discovery, it took only a glance until they were adapted and used for targeted modification of the genome (Jinek et al., 2012). Since the CRISPR-Cas9 system from *Streptococcus pyogenes* was used for GEd, more and more variations have been used for optimized targeted genome modification (Huang and Puchta, 2021).

All SDN systems in sensu stricto use the same mode of action: once present in a cell by insertion/expression and/or transfection, the SDN is capable of cutting the genome at a targeted site and introduces a double-strand break (DSB). This DSB is repaired by the cellular repair mechanisms either by nonhomologous end joining (NHEJ) or by homology-directed repair (HDR). NHEJ can be an error-prone process and due to continuous cutting of the target site (if repaired in the correct way) mutations may occur; besides deletions of various sizes, this includes insertions as well as substitutions. The presence of a sequence that is homologous to the cut region (with or without changes) can be used to trigger the HDR pathway and corresponding modifications mirror the presented template. However, the HDR pathway can also be used to introduce allochthonous (transgenes) or autochthonous sequences (cisgenes) and create a novel combination of genetic material. Furthermore, when two (or even more) independent sites (loci) are targeted within one genome (chromosomes) larger deletions or even chromosome rearrangements can be induced that otherwise rarely occur naturally during DSB-repair (Beying et al., 2020). Thus, GEd using SDNs can be seen as multiple classes:

1. the induction of single point mutations or InDels (SDN-1),
2. short intended insertions or editing of a few base pairs using an external DNA-template sequence (SDN-2)
3. the insertion of longer strands (SDN-3) of allochthonous (transgenes) or autochthonous sequences (cisgenes)

Besides DSB induction by SDNs further achievements have been made in the last years, including techniques that do not introduce a DSB, but rather create a single-stranded nick (nickases) or just modifying nucleotides (Base Editing) or introducing a reverse transcribed guideRNA into the genome (Prime editing). Furthermore, also techniques that alter the epigenome of an organism have been created by adapting SDNs. The latest achievement, which has not been shown in plants so far is the editing of the transcriptome rather than the genome by a novel type of nucleases.

Many countries legally categorize GEd approaches using the SDN1/2/3 system or similar tiers but also aberrations from this categorization can be seen in some jurisdictions. However, only a few countries have released novel regulations covering specifically GEd (and related technologies). Some countries already made amendments to their current regulations and still the majority of countries are stuck in debates on whether and how to regulate GEd and other novel techniques which are able to alter genomes (Menz et al., 2020). However, technological developments in this field are very fast and some lately released regulations are already outdated, as some techniques (e.g. Base Editing) are not captured by the regulations. It will be one of the major tasks in the coming years to come up with harmonized regulatory regimes, that are flexible enough to cope with technical developments and ensure legal certainty for all, developers, producers, traders as well as consumers. With this publication, we are clearly aiming to give an update on recent developments happening in the field of regulatory policies around the globe since the end of 2020. We are not presenting any regulation which was released before as there are publications, including Some of our own, presenting these developments, e.g. in South America, the United States, and other major agricultural countries (Menz et al., 2020; Schmidt et al., 2020; Turnbull et al., 2021).

## Recent policy activities in the EU

In July 2018, the European Court of Justice ruled that plants derived by mutagenesis techniques are to be considered as GMO and that techniques developed and established later than 2001—like GEd—demand the application of all obligations for the approval of transgenic organisms. The increasing number of advanced research on Ged applications in many crops and deliberate regulations of Ged plant products worldwide (e.g. Menz et al., 2020, this paper), and with envisaged (Jorasch, 2020) and first market releases, initiated a policy debate within the EU how to address law enforcement facing international trade as well as about the proportionality of regulatory demands.

The Council of the European Union asked the European Commission to conduct a study on the impact of the ruling. This study took into account the state-of-the-art knowledge and the views of the EU countries and stakeholders. It was published by the Commission end of April 2021 (EC 2019; 2021a). The findings of the study were the starting point for a further policy initiative by the Commission to explore the necessity and perspectives to update the GMO regulations with regards to GEd and cisgenesis in plants (only). A primary step was the so-called Inception Impact Assessment (IIA) outlining objectives and a roadmap toward the decision on a legislative action (published in September 2021, EC, 2021b). This IIA concluded that the given EU regulation on GMOs bears legal uncertainties, disproportionate or inadequate requirements, enforcement challenges and may hinder contribution of GEd to a more sustainable and resilient agricultural system (regarding Green Deal, Farm to Fork Strategy, Biodiversity strategy). The future legislation shall maintain a high level of protection, enable access to benefits, enhance the competitiveness of the EU and ensure the consistent functioning of the internal market. The key policy options for further reconsideration relate to a proportionate risk assessment and approval requirements of GEd and cisgenetic plants, sustainability analysis, appropriate traceability, and labeling provisions that are implementable and enforceable. In the course of the public consultation about the IIA in October and November 2021, the Commission clarified that it is not intending to deregulate GEd.

Until spring 2022, an in-depth impact assessment with a detailed suggestion for a legal initiative will follow with a public consultation prior to further legislative steps. Such impact assessment will explore economic, social, and environmental impacts of a revised or amendment regulation. Issues on administrative burdens and with regard to fundamental EU rights are to be considered.

There are two potentially interrelating policy actions that are linked with the legislation of GEd plants and worth following and tracking the further discussions. There is the “Sustainable food system framework initiative” (EC, 2021c) that aims to make the EU food system sustainable and to integrate sustainability into all food-related policies based on a respective regulation. The topic relates to the discussion about the criteria for sustainability of GEd plants. The initiative for the “Revision of the plant and forest reproductive material legislation” (EC, 2021d), is less observed in the broader public, but intensely discussed in the breeding sector. It addresses issues about the market introduction of plant reproductive material, IPR, and accessibility that are also key topics in the European debate about GEd plants.

In **Hungary,** which is one of the strongest opponents of genetically engineering in the EU, it came somehow as a surprise that the Ministry of Agriculture as well as financial, scientific, and agricultural organizations support “non-transgenic genome editing.” Since the ECJ ruling, the ministry is somewhat in a passive mode and nothing has been released officially but statements, e.g. from the Hungarian minister of agriculture that the EU GMO legislation should be revised. If and when an official position will be released is to date still uncertain.

### Other European and North American countries

In **Switzerland,** the association “Varieties for Tomorrow” which is composed of various actors from the Swiss agriculture and food industry called for an open and differentiated approach to NGT in plant breeding. Furthermore, the Science Commission of the States voted for the first time in favor of GEd. The Commission demands an exemption of GEd if no foreign DNA has been introduced into the plant (WBK-S, 2021). The current moratorium for commercial GM plant cultivation that is in place in Switzerland shall be prolonged for another 4 years but plants without foreign DNA shall be exempted. The **United Kingdom** released new plans “to unlock the power of gene editing to help farmers to grow more resistant, more nutritious and more productive crops” on September 29, 2021. The plan is divided into several steps. In a first step, the government wants to use the existing Environmental Protection Act 1990 to lay a Statutory Instrument by the end of 2021. The aim is to make research and development easier for plants that have been produced by genetic technologies where the resulting genetic changes could have been developed using traditional breeding methods. This enables research performing field trials with GEd plants without obligation of an extended risk assessment. However, scientists will continue to be required to notify Department for Environment, Food and Rural Affairs (DEFRA) of any research trials. As a second step, DEFRA aims to review the regulatory definitions of genetically modified organisms, to exclude such ones produced by GEd and other techniques if the product could have been developed using traditional breeding methods. GMO regulations would continue to apply if gene editing introduces DNA from other species into an organism. Furthermore, appropriate measures enabling GEd products to be brought to market, including consumer choice and traceability, will be considered in the near future in England (DEFRA, 2021a, 2021b, 2021c). In January 2022, the parliament released a Post Note on Genome Editing clarifying that the government is proposing that genome-edited crop plants are exempted from GMOs regulations, provided the genetic changes could occur naturally or via existing conventional breeding techniques. This will be achieved in two steps: the first step, which enables field trials, is already being laid by a statutory instrument; the second step will change the definition of a GMO in England and exclude GEd from it. For the registration of plant varieties, a new category will be proposed (UK-parliament, 2022)

**In Canada,** there is no separate GMO regulation, but there is a regulatory regime dealing with novel foods and with respect to risk assessment obligations with new substances notification. Following from a consultation from May 19 until September 16, 2021, Canada released a complementary draft guidance to Part V of the Seeds Regulations (Regulations Respecting the Quality of Seeds Including Seed Potatoes, and the Testing, Inspection, and Sale Thereof) for clarifying the regulatory status of seeds from plants derived with modern breeding techniques. Seed that is not substantially equivalent to the seed of that species that is already present in Canada, in terms of its specific use and safety for the environment and human health, is subject to Part V, and must be authorized before releasing into the environment. The guidance applies to all plants intended for release into the Canadian environment, including agricultural crops independent of the various technologies that may be used in the development of a plant. The draft guidance states:

Virtually all plants developed by conventional breeding techniques qualify for an exemption from Part V, on the basis of being substantially equivalent to the lines they are derived from. Similarly, genetic changes that do not include foreign DNA will, for the most part, resemble conventional breeding outcomes, and will also qualify for an exemption. The CFIA recognizes that **gene editing techniques can introduce genetic changes that are comparable to conventional breeding outcomes, and will also qualify for an exemption.** Plants derived from populations that have been previously grown in Canada qualify for an exemption, provided that they do not present new risks to the environment. Plants previously grown in Canada include those that were present prior to 1996 when Part V came into force, as well as those that were authorized after 1996. (emphasis added)

The presence of foreign DNA in the final product triggers the novelty aspect of the Canadian regulatory regime and so for the Seed Regulation. The DNA that encodes the gene editing components (e.g. CRISPR Cas protein(s) and associated guide RNAs) are considered to be foreign DNA. If these sequences are removed from the final products by using plant breeding and selection, it is exempted, if no other point of the regulation is met. If the sequences stay within the plant material or if the plant species is new to Canada, it is still subject to Part V. If a plant is exempt from Part V, there is no requirement to submit information to the CFIA. The plant can be released in Canada, subject to any other applicable requirements; if needed the CFIA will issue an exemption opinion letter (CFIA, 2021).

## Policy activities outside the EU

### Africa

**Nigeria** has authorized guidelines on gene editing through the National Biosafety Management Agency (NBMA) in December 2020 (NMBA, 2020). The Government of Nigeria sees science and technology (including “molecular breeding techniques”) as a major driver of agricultural productivity and as part of the solution to provide food security and fight malnutrition in Nigeria and Africa as a whole. NBMA aimed to introduce adequate regulation that will ensure that products of GEd do not cause harm or prevent any adverse effect to the environment, humans, and plants. The released guidelines are directed to all “person(s), institutions or bodies wishing to carry out gene editing as it relates to **plants**, **animals,** and **microorganisms** ranging from containment, confined field trial, multi-locational trial, commercial/general release and imports intended for direct use as food or feed, or for processing". Nigeria has adopted a case-by-case approach to regulate GEd and products thereof. Products of GEd that result in a “novel” combination of genetic material or contain recombinant DNA such as nuclease gene, present in the final product (InDels are not seen as novel combination) will be classified as GMO and regulated as such. However, products, which do not contain “recombinant DNA,” or the recombinant DNA that has been removed in the final product is not seen as GMO and a non-GMO regulatory classification is applied. Nonetheless, every applicant wishing to carry out gene editing in Nigeria has to approach NBMA, which will come back to the applicant with feedback within 21 d.

**South Africa** which was long time undecided on how to deal with GEd has announced its regulatory approaches for “New breeding technologies” through a public notice after a discussion on the GMO status of “NBT products” on October 27, 2021 (Republic of South Africa, 2021). The executive council of South Africa has concluded that for “NBTs” the same risk assessment framework should apply as for GMOs based on the definition of a GMO in the South African GMO Act. Based on this decision, the application forms were updated and products of “NBTs” are seen as a GMO regardless of the type.

In contrast to this, **Kenya** has recently released its guidelines as well. The Kenyan guidelines rely on a case-by-case evaluation based on the presence of transgenes and are comparable to the ones which are in place in Nigeria including deregulation of cisgenesis and in cases where foreign DNA is absent (“all deletions/knock outs”). Furthermore, processed products whose inserted foreign genetic material cannot be detected (e.g. processed Oils) will also be excluded, the same holds true for “conventional” breeding methods, e.g. mutagenesis, polyploidy, and haploidy (Entine et al., 2021; NBAK, 2022). The applicant in Kenya will receive feedback within 30 d. Kenya has already approved five GEd events including three which confer resistance toward plant–pests.

Some other countries in Africa are still in the debate on GEd and haven’t come up with regulations so far. Most advanced drafts were prepared by **Eswatini and Burkina Faso** (ASF, 2021; NEPAD, 2021). Burkina Faso is running experiments with genome-edited rice (*Oryza sativa*) which is resistant to bacterial blight. Other countries in Africa like **Mali, Senegal, Gambia, Niger, Mauritania,** and **Guinea-Bissau** do have a regulatory system for GMOs in place or are in the process of releasing, when and whether this will specifically be adapted to GEd is uncertain, but discussions in Africa are being held intensively. However, the trend for current and future regulations in Africa tends toward open regulations just as in South American countries.

### Asia

**South Korea** has informed the WTO in a short statement on the introduction of a preliminary review system for low-risk LMOs, covering “LMOs created through modern biotechnology” under Article 2 (Definition) of the LMO Act. In the short statement, South Korea notifies that “low-risk LMOs in which foreign gene has not been introduced to the final product shall be exempted from risk review and approval on the import, production and contained use.” Further details will follow adoption of the LMO policy. A formal statement is expected in late 2022, clarifying which kinds of changes will be excluded from the regulation (WTO, 2021).

In **Japan,** amendments have been made to the current guidance on GEd in December 2020. The Ministry of Health, Labour and Welfare (MHLW) amended the handling procedures for food and food additives that are derived from cross-breeding varieties which have already been notified to MHLW, and these products are no longer subject to the MHLW consultation process. In April 2021, the Ministry of Agriculture, Forestry and Fisheries (MAFF) approved a proposal to amend the Feed Safety guidelines for handling of “genome edited feed and feed additives developed by crossbreeding already notified genome edited organisms with other allowed varieties.” In the past, products derived from crossbreeding of genome-edited varieties had to undergo a consultation with MAFF. These guidelines have been adapted on April 20, 2021 notifying that no prior consultation is required anymore of crosses of “conventional varieties etc.*… with varieties that have been notified as genome edited feed.” The etc.* category includes, but is not limited to products notified to MAFF as genome-edited, genetically engineered, etc. The same holds true for MHLW.

**Indonesia** drafted a regulation on CRISPR-based GEd and other GEd techniques in early 2021. According to the draft, GEd products will be regulated as GMOs when they contain a novel combination of DNA (“from outside the taxon”) or if foreign DNA is present in the final product. If this is not the case the products will be deregulated, and further details should be published later in 2022 (Prasetya and Nugroho, 2021).

**The Philippines** released a resolution in late 2020 declaring that products of “innovative breeding techniques” such as GEd are going to be regulated under GMO law in the case of novel combination of genetic material, which is not possible to achieve through conventional breeding. In the other cases, products should be regulated as non-GMO or conventional. The resolution is going to be set in a new regulation that will clarify further details. The regulation is expected in the mid-2022 (USDA, 2021a).

**India** has recently defined regulations for products of innovative technologies such as GEd. A draft document on a proposed regulation has been published and commented by the public by July 2020. The draft stated that products of SDN1 and SDN2 GEd approaches that do not carry or involve exogenous DNA and are comparable to naturally occurring events should be exempted from the Indian GMO legislation. However, an appropriate tiered-based risk assessment is foreseen categorizing GEd into three categories. The first category in which single or few base pair edits/deletions should be addressed, an assessment is made to confirm targeted edits as well as the absence of any biological relevant off-target genomic changes. Phenotypic equivalence will be checked if necessary on a case-by-case basis. The second category that covers targeted few/several base pair edits an assessment will be supplemented with phenotypic equivalence and trait efficacy through appropriate contained and/or confined field trials. The last category addresses products harboring targeted edit(s) synthetic/foreign DNA. The assessment is the same as for traditional GMOs. On March 30, the Indian Ministry of Environment, Forest and Climate Change accepted in many parts of the proposed regulation and exempted genome-edited plants of the category SDN1 and 2 from the Indian GMO regulation (MST, 2020; DEFC, 2022).

**China** has released guidelines for the safety evaluation of gene-edited plants for agricultural use. These guidelines cover all products of GEd that have not introduced exogeneous genes (all except SDN3 approaches). For products that have introduced exogenous genes the classical GMO guidelines are applicable. The guidelines for GEd provide four requirement categories, based on the risk profile of the target trait. The first category does not increase the risk of environmental and food safety, the second may increase environmental risks, the third may increase food safety risks and the fourth may increase both risks. For each category, different requirements need to be provided for production and or import. There are some general requirements that need to be provided in every category: (1) molecular characterization of the plant including targeted gene-related data, gene editing method applied, data on the targeted gene edit (on target data), potential vector sequence residue, and off-target analysis, as well as (2) genetic stability of the edit and the trait in at least three generations. Additionally, the need for environmental and/or food safety assessment needs to be provided if necessary. These requirements are in line with the ones requested in the guidelines for the safety evaluation of GMOs (USDA, 2022). However, the guidelines do not indicate how to classify a product into one of the four categories nor to which extent data have to be provided. This will presumably be claimed on a case-by-case basis, but no official statement on this has been released. Clarification will be granted after the first decision has been made in China.

Other countries in South-East Asia are still in debate including **Bangladesh**, **Vietnam,** and **Taiwan** (Schmidt et al., 2020; USDA, 2021b, 2021c). **Taiwan’s** food and drug administration is working with research institutes in Taiwan to draft a regulatory guidance for innovative biotechnologies including gene editing. However, a public draft is still forthcoming, but considerations concerning risk assessment and management are being prepared. **Vietnam** is amongst other countries (e.g. United States, Canada, Uruguay, etc.) a supporter of the International Statement on Agricultural Application of Precision Biotechnology submitted to the WTO, but when and how a draft will be published is still open (WTO, 2020).

## Perception of GE in society

Aside from a shift and the clarification of the legal status of GEd products in many jurisdictions, as described above many countries have clarified their legal status of GEd products in the last 2 years. Besides this, some other countries, especially in jurisdictions that haven’t decided yet, tried to image the public perception of NGTs in their respective countries through official surveys. The Norwegian collaboration GENEinnovate performed a sophisticated consumer survey to elaborate a more precise understanding of how consumers are informed and how is their opinion, with respect to GE (Norwegian Biotechnology Advisory Board, 2020). GENEinnovate is a collaboration of breeders, companies the Norwegian University of Life Sciences, and the Norwegian Biotechnology Advisory Board and therefore represents a broad collection of stakeholders. The results of the survey showed that there is more than just like or dislike of GE in the Norwegian population. Regarding the knowledge of consumers, the vast majority (96%) have heard about genetically modified foods but less than 50% have ever heard about GE. Regardless of this, the general attitude of more than 50% of the consumers (up to 70%) was positive with respect to using GEd for breeding purpose that result in traits with a clear sustainability or societal benefit (i.e. reducing pesticide use or climate adaption of plants). In contrast to this, if GEd is used for traits that are of no societal benefit (e.g. changing the appearance of a product), the consumers are more negative. In summary, the survey showed that the consumer attitude is nuanced and that the important fact is the new trait and not the technique used to create it. Furthermore, there is a need for knowledge building about new genetic techniques in the public.

A very similar survey was conducted in 2020 by The Swedish Technology advisory board. This report used the questions from the Norwegian survey and adapted it to the Swedish population. The report is not published so far but it showed that, as in Norway, almost half of the Swedish consumers never heard about gene scissors or GE. Again as in Norway, the main result of this survey was that there is a positive attitude of more than 60% toward GE plants if the trait would contribute to environmental or societal benefits. If this is not the case the attitude is negative (The Swedish Gene Technology Advisory Board, 2022).

Another survey was conducted in 2021 by The Food Standards Agency as a consumer poll (The Food Standards Agency, 2021). The key findings of this poll were that consumers in the United Kingdom find GEd more acceptable than GM and in general GM or GE applied to plants more acceptable than either of them applied to animals. As in the Norwegian survey, more informed consumers were more accepting of GE food than less informed ones. Interestingly, most consumers felt it appropriate to regulate GE and GM differently. Another claim of the poll was that United Kingdom consumers demand a transparent labeling if GE food reaches the market. In parallel to this consumer poll, the DEFRA held a public consultation (January 7 to March 17, 2021) to collect views regarding the regulation of genetic technologies in England. As outcome of this consultation, DEFRA as first step wants to alleviate the burdens on developers doing research and development using genetic technologies. Field trials will be possible without risk assessment but still under notification duty. As a second step, DEFRA wants to elaborate an amendment regarding the regulatory definitions of a GMO in a way that organism “that could have been achieved through traditional breeding or occur naturally” but have been produced by genetic technologies are exempted from GMO regulation (DEFRA, 2021c).

Eventually, The Australian National University performed a systematic literature review (Grant et al., 2021). This review highlighted that the existing literature is limited, riven by gaps, and often methodologically framed or biased in other aspects. A reasonable number of the reviewed literature focused solely on the consumer’s “Willingness to pay” and neglected other aspects. Nevertheless, the key findings in the systematic literature review showed that there is a slightly more positive consumer attitude regarding food produced from GE techniques than toward older forms of genetic modification (Grant et al., 2021).

## Conclusion

**Figure 1:**
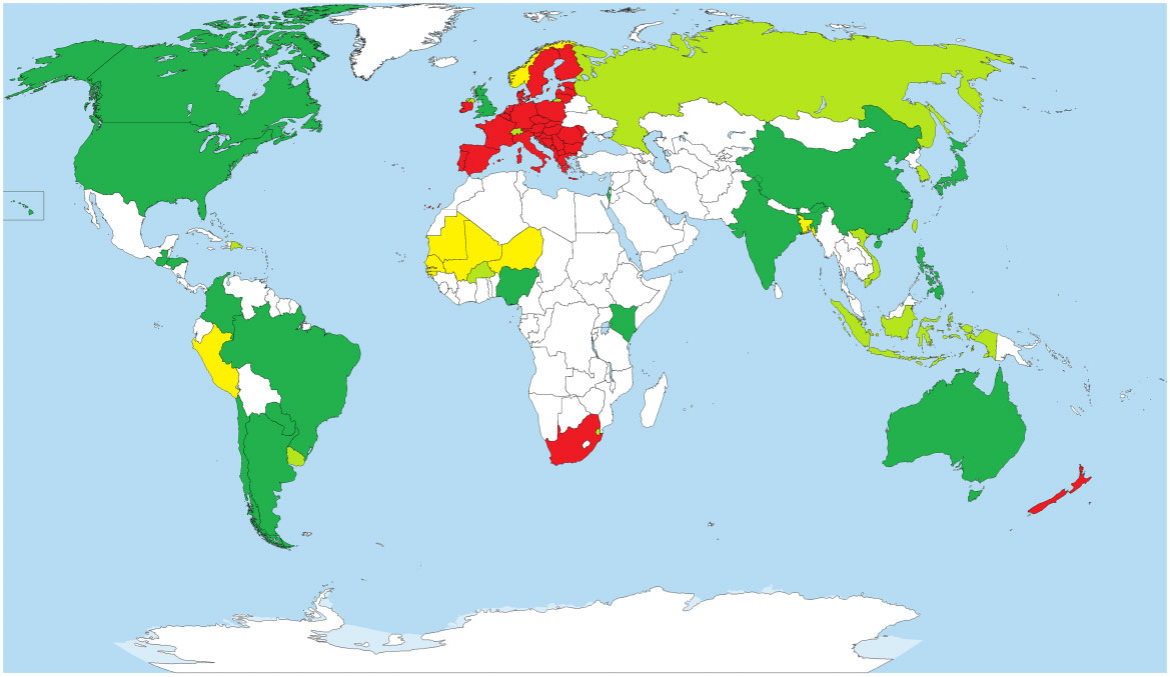
The regulatory landscape on a global schema. Color code: Dark green: legislation open toward GEd, light green: open legislation or positive statement being prepared, yellow: discussion ongoing with no decision yet. Red: strict GMO regulation for GEd products. White: no discussion on GEd or no information available.

Renovation of GMO regulatory regimes evolved rapidly in the last 2 years, especially in the beginning of 2022. With China and India clarifying their policy on GEd and opening up for a cultivation of GEd plants, the two countries with the highest population worldwide have made a clear step toward the use of GEd in agricultural products. Looking at the 10 countries with the highest population worldwide 9 out of 10 (except Pakistan) have paved a way, or stated intentions to open up for easy use of genome-edited organisms (essentially plants) in commercial agriculture. Many of those countries are also among the top agricultural producers in the world (especially China, United States, India, and Brazil). However, the debate to deregulate GEd is still controversial in other parts of the world, especially in the European Union and associated countries (see “Outstanding questions” section). The EU is an important region for the import of agricultural products worldwide and therefore an important trade partner to many countries. Several of those already have a GEd regulation in place which allows exemptions of some GEd products from strict GMO regulations including labeling. This situation is contrary to the legislation in Europe, and the situation may worsen, as clear detection and identification of such products in goods is currently not possible without technical details (unique analytical reference data), and will not be possible in the near future (Grohmann et al., 2019; Weidner et al., 2022). In the past, many countries remained on hold waiting for Europe to make a move toward a clear regulation (see Sprink et al., 2016). However, the situation seems to have changed as many countries esp. in Africa seem to open up and also members of the EU (Hungary) and associated countries (Switzerland, United Kingdom, Norway) discuss regulatory options for gene editing or a pledging for an exclusion of some products of GEd from strict GMO regulations. The major question in the next year will be whether and how far the EU will ease the obligations for genome-edited plant products. The more countries deregulate GEd organisms and when more products will strive to international markets, the pressure is rising in the undecided countries to clarify the regulatory status and enforcement measures. However, the way to a globally harmonized system is still a long way to go since different countries that allow GE have somewhat differing regulations or amendments in place and some countries haven’t decided, yet (Figure 1).

**OUTSTANDING QUESTIONS**

- How will products of GEd be identified in the future as the cause of a mutation cannot regularly be proven?
- Will the not solved detection and identification issue have a detrimental effect on global trade and accessibility of breeding material?
- How will a future regulation of GMOs and/or GEd in the EU look like and when will it be released?
- Will it be possible to achieve a harmonized global regulation in the future?

*Conflict of interest statement*. The authors declare no conflicts of interest.

## Notes

T.S., R.W., and F.H. wrote the manuscript.

The author responsible for distribution of materials integral to the findings presented in this article in accordance with the policy described in the Instructions for Authors (https://academic.oup.com/pphys/pages/General-Instructions) is Thorben Sprink.
